# Integrating the GSA KAER Toolkit Into Practical Training for Primary Care Clinics

**DOI:** 10.1093/geroni/igab046.1336

**Published:** 2021-12-17

**Authors:** Barak Gaster, Jaqueline Raetz, Basia Belza, Monica Zigman Suchsland, Judit Illes, Benjamin Olivari, Lisa McGuire, Annette Fitzpatrick

**Affiliations:** 1 University of Washington, Seattle, Washington, United States; 2 University of Washington, Seattle, University of Washington, Washington, United States; 3 The Gerontological Society of America, Washington, District of Columbia, United States; 4 Centers for Disease Control and Prevention (CDC), Atlanta, Georgia, United States; 5 CDC, Atlanta, Georgia, United States

## Abstract

Education is central to driving change in clinical practice. First, primary care providers and their clinic team members need to understand why detecting cognitive impairment is important, how it can be done efficiently, and what the next steps in referral and management are. To engage primary care clinics in this change process, we developed a continuing education intervention, based on the KAER Model, using a live video format. Four evidence-based, 45-minute training modules presented core knowledge skills, including how to have difficult conversations, which are essential to diagnosing cognitive impairment. To overcome the obstacles to doing so in primary care, our team relied on a deep understanding of busy primary care practice. With a combined 35 years of direct experience in primary care, our collaborative interdisciplinary team was able to use the KAER Model to develop a highly acceptable intervention for primary care.

